# Proteome alterations associated with transformation of multiple myeloma to secondary plasma cell leukemia

**DOI:** 10.18632/oncotarget.14294

**Published:** 2016-12-27

**Authors:** Alexey Zatula, Aida Dikic, Celine Mulder, Animesh Sharma, Cathrine B. Vågbø, Mirta M.L. Sousa, Anders Waage, Geir Slupphaug

**Affiliations:** ^1^ Department of Cancer Research and Molecular Medicine, Norwegian University of Science and Technology, NTNU, Trondheim, Norway; ^2^ Present address: University of Utrecht, Utrecht, Holland; ^3^ PROMEC Core Facility for Proteomics and Metabolomics, Norwegian University of Science and Technology, NTNU, Trondheim, and the Central Norway Regional Health Authority, Stjørdal, Norway; ^4^ Department of Hematology, Department of Medicine, St. Olav's Hospital, Trondheim, Norway

**Keywords:** multiple myeloma, secondary plasma cell leukemia, super-SILAC, quantitative proteomics

## Abstract

Plasma cell leukemia is a rare and aggressive plasma cell neoplasm that may either originate de novo (primary PCL) or by leukemic transformation of multiple myeloma (MM) to secondary PCL (sPCL). The prognosis of sPCL is very poor, and currently no standard treatment is available due to lack of prospective clinical studies. In an attempt to elucidate factors contributing to transformation, we have performed super-SILAC quantitative proteome profiling of malignant plasma cells collected from the same patient at both the MM and sPCL stages of the disease. 795 proteins were found to be differentially expressed in the MM and sPCL samples. Gene ontology analysis indicated a metabolic shift towards aerobic glycolysis in sPCL as well as marked down-regulation of enzymes involved in glycan synthesis, potentially mediating altered glycosylation of surface receptors. There was no significant change in overall genomic 5-methylcytosine or 5-hydroxymethylcytosine at the two stages, indicating that epigenetic dysregulation was not a major driver of transformation to sPCL. The present study constitutes the first attempt to provide a comprehensive map of the altered protein expression profile accompanying transformation of MM to sPCL in a single patient, identifying several candidate proteins that can be targeted by currently available small molecule drugs. Our dataset furthermore constitutes a reference dataset for further proteomic analysis of sPCL transformation.

## INTRODUCTION

Plasma cell leukemia (PCL) is a rare and aggressive lymphoproliferative disorder characterized by high levels of malignant plasma cells in the peripheral blood [1]. It can manifest either as *de novo* (primary) pPCL or as a secondary transformation (sPCL) of multiple myeloma (MM) and gene expression profiling suggests that the two forms constitute separate molecular entities [2]. The overall incidence rate in Europe of all PCL is approximately 1 case per 2.5 million persons/year [3] and of these generally 30 - 40% constitute sPCL [4]. sPCL is associated with poor prognosis and there is currently no standard treatment due to the lack of prospective data on treatment regimens and outcome in large trials. The mechanisms whereby MM transforms to sPCL remain elusive, but different secondary genomic events accumulating upon primary events present at the MM stage likely contribute [5]. Primary events commonly seen in MM are trisomies and IgH translocations with *CCND1* [6], *CCND3* [7], *MMSET/FGFR3* [8], *cMAF* [9] and *MAFB* [10]. Examples of secondary events are deletion or inactivation of *TP53* and activation of proto-oncogenes *c-MYC*, *N-RAS* and *K-RAS* [11], deletion of PTEN [12] and Rb [13]. Interestingly, unlike MM, monoallelic or biallelic inactivation of TP53 does not correlate with survival [14, 15], suggesting ubiquitous targeting of the p53 pathway in sPCL [16]. Immunophenotypic profiling of PCL versus MM cells suggests that modulated expression of some surface antigens might contribute to the escape from the bone marrow environment and also from immunological surveillance, including down-regulation of CD11a/b and CD18 [17] and CD56. Moreover, CD28 is more frequently expressed in sPCL than in MM, consistent with the observation that increased CD28 expression in MM plasma cells correlates with increased proliferation and progression [18]. Finally, a longitudinal whole-genome sequencing study in a single patient progressing from MM to sPCL identified several loss-of function mutations only occurring at the final sPCL stage, including *ZKSCAN3* and *RB1*, which, together with deletion of *TP53* could lead to dysregulation of cell-cycle checkpoints [19].

Although the above studies have provided some clues towards understanding factors driving the progression from MM to sPCL, to the best of our knowledge no attempts have been made to study alterations at the whole-proteome level accompanying the transformation. Such studies are by no means trivial. sPCL is very rare, thus sufficient samples for robust statistical evaluation will be extremely hard to obtain. Moreover, by escaping the bone marrow into peripheral blood the malignant plasma cells will likely adapt to the novel environment by modulating expression of several proteins. Deciphering drivers and bystanders may thus be a challenging task. To initially address these questions we here present a super-SILAC [20] quantitative proteome analysis of purified malignant plasma cells obtained from a single patient at the MM and the sPCL stages. SILAC (stable isotope labeling with amino acids in cell culture) is an accurate and reliable quantitative proteomics method that detects differences in protein abundance among samples using non-radioactive isotopic labeling [21]. Reference cells are labeled through the incorporation of “heavy” versions of essential amino acids in the cell populations and mixed early in the sample preparation phase together with cells of interest and are analyzed together by LC-MS/MS (commonly ^13^C_6_^14^N_2_-lysine and ^13^C_6_^14^N_4_-arginine are utilized, which produce a mass difference of 8.0142 Da and 10.00827 Da, respectively, for each tryptic peptide). The SILAC approach is mainly limited to proliferating cells that can be metabolically labeled with heavy amino acids. For studying e.g. patient samples, this can be circumvented by using a heavy-labelled protein mixture as internal standard. In this approach, denoted super-SILAC, a mixture of proteins extracted from several SILAC-labeled cell lines serve together as the spike-in standard. By spiking the same amount of SILAC-labeled standard in each sample, a precise relative comparison of protein levels between samples can be indirectly obtained. Super-SILAC holds tremendous potential in e.g. clinical diagnosis. This was demonstrated by Deeb et al [22], who were able to segregate two histologically indistinguishable subtypes of diffuse large B-cell lymphoma (DLBCL), activated B-cell-like (ABC) and germinal-center B-cell-like (GCB) subtypes, by employing this method. A dataset has been created using Orbitrap Elite MS combined with a super-SILAC experimental setup. This dataset is a first attempt to shed light on the transition from MM to sPCL from a proteomic point of view and can be used as future reference in the ongoing research on MM and sPCL.

## RESULTS AND DISCUSSION

### Generation of a multiple myeloma super-SILAC library

**Figure 1:**
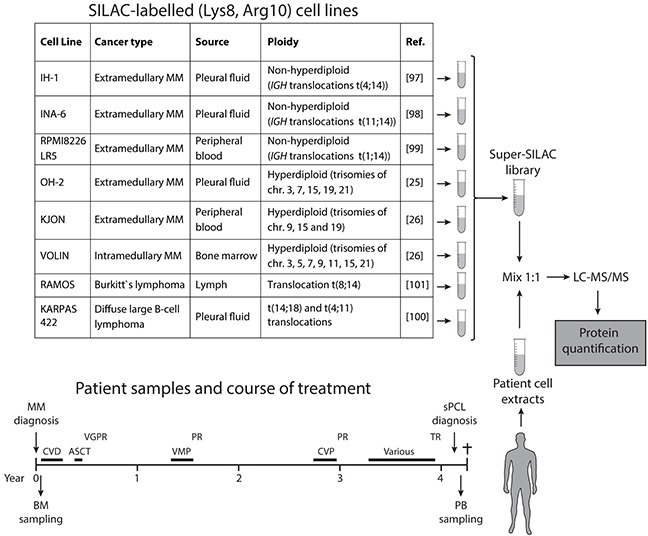
Schematic illustration of the super-SILAC workflow The table shows the cell lines included in the super-SILAC library, their sources as well as major genetic characteristics. Below is shown the timeline of the patient treatments and the collection of bone marrow (BM) and peripheral blood (PB) samples. CVD; cyclophosphamide, bortezomib, dexamethasone, VMP; bortezomib/melphalan/prednisone, CVP; cyclophosphamide, bortezomib, prednisone, VGPR; very good partial remission, PR; partial remission, TR; treatment resistant.

To accurately quantify proteome differences between multiple myeloma and sPCL, we generated a super-SILAC cell library consisting of three non-hyperdiploid (IH-1, INA-6, RPMI8226-LR5) and three hyperdiploid (OH-2, KJON, VOLIN) MM cell lines, as well as two B-cell lymphoma cell lines (RAMOS, KARPAS-422). Importantly, about 55-60% of multiple myelomas are hyperdiploid with 48-74 chromosomes. They often contain trisomies of the odd numbered chromosomes 3, 5, 7, 9, 11, 15, 19, and 21 and less frequently *IGH*-translocations. However, nearly all available MM cell lines are derived from non-hyperdiploid myelomas, and would thus not fully represent the disease [23, 24]. We have been able to establish three multiple myeloma cell lines from hyperdiploid multiple myeloma patients, named OH-2 [25], KJON and VOLIN [26]. Of these, OH-2 and VOLIN only reached 71% and 80% incorporation of heavy amino acids, respectively, whereas all the other cell lines reached 94% incorporation or above. Notably, whereas near complete incorporation of heavy label is important to avoid skewing of quantitative data in standard SILAC experiments, this is less of a problem in super-SILAC since an equal amount of the heavy library is mixed with every patient sample. The final output represent “ratios of ratios” and there is little risk of introducing false positives (erroneously high differential expression) due to non-complete labelling. Despite suboptimal incorporation of heavy label in OH-1 and VOLIN, we thus decided to include them in the library to get equal representation of hyperdiploid and non-hyperdiploid cell lines. We included the two B-cell lymphoma cell lines since they express high levels of activation-induced deaminase (AID) [27], and which was not quantified in any of the MM cell lines used in the library. An AID/APOBEC mutational signature was recently shown in 3.8% of myeloma cases and linked to deregulated MAF, MAFB and MYC and poor prognosis [28]. Finally, the non-hyperdiploid cell line RPMI8226-LR5 was included as a representative of a drug-resistant cell line, grown under continuous melphalan exposure [29]. The cell lines employed in the super-SILAC library, their characteristics, and the experimental outline are presented in Figure 1.

**Figure 2:**
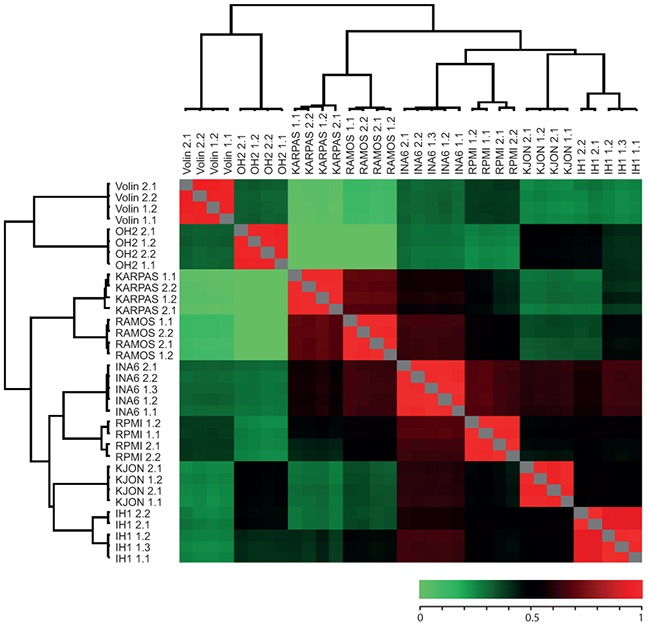
Heat map of Pearson showing reproducibility between replicates as well as similarity between certain cell lines Numbers succeeding each cell line indicate biological and technical replicates. The color bar represent the corresponding correlation coefficients.

To further characterize the cell lines we prepared duplicate protein extracts from each of their non-labelled versions and individually spiked these 1:1 with the super-SILAC mixture. Each of the combined extracts was then analyzed by LC-MS/MS in at least two technical replicates as described in Materials and Methods. Joint analysis of the result files in MaxQuant identified a total of ~5270 different protein groups from the cell lines (on average ~3100 per cell line). To compare the cell line proteomes we quantified all measurements against each other based on the ratios to the super-SILAC mix and calculated their correlation coefficients. Further, we performed unsupervised hierarchical clustering of the quantified proteins using normalized SILAC-ratios to reveal the degree of pairwise differences in protein expression patterns at a global level (Figure 2; the complete list of pairwise correlation coefficients and P-values can be found in Supplementary Table S1). In each case the rows and columns of the matrix of coefficients co-clustered the replicates (biological and technical) in a tight fashion (Figure 2), indicating good reproducibility [20]. Moreover, the lack of significant overlap between the cell lines indicated that the library represented a broad coverage of the MM cancer proteome. Notably, this analysis also indicated that the two cell lines having the most pronounced hyperdiploidy, OH-2 and VOLIN, were found to be dissimilar from the rest of the cell lines, and segregated even further away from the other MM cell lines than the two B-cell lymphoma cell lines RAMOS and KARPAS422. A potential effect of non-complete labelling to the segregation of OH-1 and VOLIN was regarded less likely, since VOLIN segregated further away from the other MM cell lines than OH-1, despite having significantly higher incorporation of heavy label. This supported that inclusion of these hyperdiploid cell lines in the library would increase coverage of potentially disease-relevant proteins.

### Comparison of the multiple myeloma and plasma cell leukemia proteomes

In our super-SILAC analysis of MM and sPCL samples an average of 3659 ± 396 (SD) proteins were identified, amongst which 2784 ± 249 were quantified by super-SILAC. To extract as much information as possible from the RAW data, label-free quantification (LFQ) light channel from super-SILAC representing the sample were also used for quantitative analysis [22, 30]. Proteins quantified in at least two out of three biological replicates were considered for further analysis. The values were log_2_ transformed in order to have a better approximation to normal distribution. Further, median of technical replicates were calculated to represent the samples in order to be less sensitive to outliers. These are presented in Supplementary Table S2. The values were compared as groups representing MM and sPCL to find differentially expressed proteins (Student's t-test). The p-values were then corrected by creating a background distribution through random assignment of values to each group. If the chance of getting a specific p-value is still < 5% after this permutation, the protein is marked as differentially expressed. This strategy has been shown to be robust to differing background distribution as empirical distributions are being created for each case. Further, it is less sensitive to number of comparisons like traditional Benjamini Hochberg correction [31].

**Figure 3:**
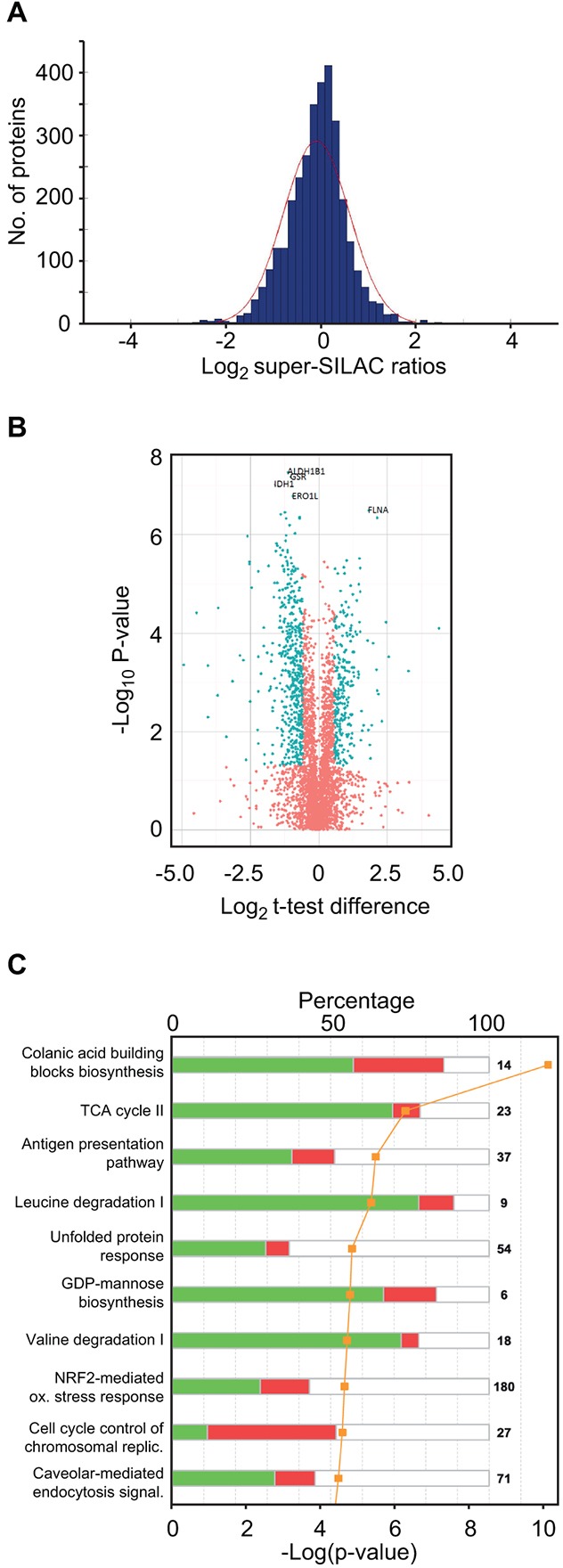
Super-SILAC quantitative profiling and pathway analysis of differentially expressed proteins **A**. Histogram of log_2_ super-SILAC ratios between proteins in the sPCL and MM samples. Negative values represent proteins with increased expression in MM, whereas positive values represent proteins with increased expression in sPCL. **B**. Volcano plot of the entire set of proteins quantified during super-SILAC analysis. Each point represents the difference in expression (Log_2_ t-test difference) between the sPCL and the MM samples plotted against the level of statistical significance. Blue dots represent proteins whose expression is significantly (t-test, p < 0.05) different in the two samples and with an absolute log_2_ t-test difference >0.58. **C**. Top 10 canonical pathways [log_10_ (p-values)] significantly changed in sPCL compared to MM, according to IPA.

Missing values were imputed following the guidelines from Deeb et al. [22] but with a down-shift of 1.5 and standard deviation of 0.5 from the total data matrix, as it reflected the noise more smoothly in the histogram representation of our data. The values from super-SILAC ratios and label-free values with and without imputation were subjected to Student's t-test with permutation-based false discovery rate (FDR) of 0.05 as implemented in Perseus software v 1.5 [32] (Supplementary Table S2). The distribution of the log_2_ super-SILAC ratios between the quantifications in MM and sPCL is shown in Figure 3A. These values show normal distribution around zero, supporting similar overall protein loads of the samples. 795 proteins were found to be differentially expressed in the MM and sPCL cells when using a log_2_ cut-off of 0.58 and p < 0.05 (Supplementary Table S2, assigned the value 1 in column G) amongst which 728 had FDR < 0.05 (assigned the value 1 in column F). Figure 3B shows a volcano plot of the entire dataset indicating proteins whose expression was significantly different (*t*-test, p < 0.05) between the sPCL and MM samples. To analyze the subcellular location of the differentially expressed proteins, IDs Gene Ontology (GO) database annotations were analyzed. Here, 36.0 % of the proteins were reported to be localized to the cytosol, 30.6% to the nucleus and 16.2% to the mitochondria. Annotation enrichments analysis [33] over these 728 expression values showed that the nuclear proteins were significantly up-regulated (median log_2_ ~ 0.61 p-value < 2E-11 with FDR < 5E-9), while mitochondrial proteins were down-regulated (median log_2_ ~ -0.76 p-value < 0.05) in the sPCL cells, indicating that the transformation was accompanied by increased nuclear and reduced mitochondrial function in general. Interestingly, reduced mitochondrial function and an increased glycolytic phenotype are often observed in tumor cells even when oxygen is present. This is commonly known as the “Warburg effect” [34] and is also associated with increased resistance to apoptosis, high invasiveness and metastasis [35]. A shift towards aerobic glycolytic metabolism was also recently associated with development of acquired resistance to Melphalan in multiple myeloma cells [29].

### Enzymes involved in protein glycosylation are down-regulated in sPCL

To search for modified biological pathways associated with transformation to sPCL, gene identifiers in the SILAC dataset were mapped in the Ingenuity® Knowledge Base (IPA) and plotted onto their canonical pathways. The ten most significant pathways are presented in Figure 3C. Here, colanic acid building blocks biosynthesis was identified as the most significantly altered pathway (p~2.24×10^−10^) where 9 out of 14 associated proteins were found to be differentially expressed and seven of which were down-regulated in PCL. Colanic acid is an extracellular bacterial polysaccharide also known as M-antigen. In humans, the nucleotide sugar building blocks are instead used in other pathways such as the glycosylation of cell surface receptors. Manual inspection of the data indeed revealed that many of the enzymes involved in the formation of nucleotide-activated sugars for glycoprotein synthesis, including of GDP-mannose, GDP-fucose, UDP-N-acetylglucosamine (UDP-GlcNAc) and CMP-N-acetylneuraminic acid (CMP-Neu5Ac) were significantly down-regulated at the sPCL stage. In the GDP-mannose synthetic pathway PMM2, GMPPA and GMPPB were 1.75-, 1.92- and 2.13-fold down-regulated (p < 0.05), respectively (Supplementary Table S2). In the first step in the further conversion of GDP-mannose to GDP-fucose, GMDS was 1.52-fold down-regulated. Moreover, in the synthesis of UDP-GlcNAc from fructose-6-phosphate, all enzymes were down-regulated, although only two of these, GFPT1 (1.73-fold) and PGM3 (2.40-fold) were statistically significant. In the further conversion of UDP-GlcNAc to CMP-Neu5Ac, NANS and CMAS were significantly down-regulated (2.3- and 2.2-fold, respectively). These results suggested that the synthesis of glycoproteins could be compromised in the sPCL cells. This was corroborated by the levels of ALG1 and ALG2, responsible for in initial transfer of GDP-mannose to proteins at the ER, which was 1.57- and 2.31-fold down-regulated, respectively. Furthermore, STT3B, the catalytic subunit of the complex transferring oligosaccharides to asparagines, was 2.5-fold down-regulated. Aberrant glycosylation of surface antigens has been associated with poor prognosis of several cancers. Among the tumor-associated glycans, sialic acids have received special attention. These negatively charged monosaccharides are typically found terminally at cell surface glycoconjugates, and commonly constitutes *N-acetylneuraminic acid* (Neu5Ac) in humans. To our knowledge, only one study has addressed the potential involvement of altered sialylation in MM cell trafficking [36]. Here, high expression of the α-2,3-sialyltransferase ST3GAL6 was associated with inferior overall survival. Moreover, knockdown of ST3GAL6 mediated reduced binding of MM cells to bone marrow stromal cells and fibronectin as well as reduced transendothelial migration *in vivo*, thus establishing a role of sialylation in MM cell trafficking. Conversely, reduced α-2,3-sialylation was recently reported in colorectal tumors compared to normal tissues, indicating that the impact of sialylation is cancer-specific. Unfortunately, ST3GAL6 was not quantified in our dataset. However, the α-2,6-sialyltransferase ST6GAL1 was 1.68-fold (p~0.001) down-regulated. Together with the reduced levels of enzymes involved in sialylation precursor synthesis, this strongly suggest that α-2,6-sialylation is reduced in the sPCL samples compared to the MM samples. The precise contribution of this as well as to the formation of other glycoconjugates to the disease progression in general must, however await further studies.

### Aberrant expression of surface receptors may contribute to evasion from the bone marrow

After their affinity maturation in germinal centers, plasma cells home to mucosal surfaces, sites of inflammation as well as to the bone marrow. This is generally accompanied by down-regulation of lymphoid tissue receptors and up-regulation of receptors for chemokines produced at the different sites. In the bone marrow, vascular cell-adhesion molecule 1 (VCAM-1) is an important mediator of plasma cell homing, through interaction with the integrin VLA-4 (ITGA4/ITGB1 dimer). Moreover, plasma cells express surface CXCR4 that migrates towards CXCL12 produced by bone marrow stromal cells as well as SDC1 (CD138, syndecan-1) that binds to fibronectin and collagen, and the adhesion molecule CD44. Intuitively, it would be expected that evasion of the malignant myeloma cells from the bone marrow is promoted by down-regulation of these surface proteins. However, no significant difference in SDC1-expression was observed in the MM and sPCL cells, whereas CD44 was 2.3-fold up-regulated (p~4.3E-05). Variants of this protein (CD44v8-v10) apparently affects the endothelial cells of small blood vessels by promoting phosphorylation of vascular endothelial cadherin, junction disruption and transendothelial migration of melanoma cells [37]. Recently, expression of CD44v9 in circulating colorectal cancer cells was also associated with poor prognosis [38], corroborating increased metastatic potential of cells harboring this variant. ITGB1, which constitutes half of the heterodimeric VLA4, was also up-regulated in the sPCL cells (1.63-fold). However, VCAM-1 is also highly expressed by newly formed blood vessels in the bone marrow (reviewed in [39]) and increased VLA4 may thus allow preferential localization of the myeloma cells to such vessels and provide a passageway to the periphery. Notably, all of the above plasma cell surface antigens are heavily glycosylated [40, 41] and it is conceivable that their perturbed glycosylation may alter their matrix interaction properties to allow bone marrow evasion. This has been particularly studied for CD44, which needs specific sialofucosylations to act as a E-/L-selectin ligand and bone homing receptor, named HCELL [42]. Correct sialylation is also a prerequisite for another selectin ligand, SELPLG. This ligand was not quantified in the super-SILAC dataset, but was quantified by LFQ in all the sPCL samples whereas it was not detected in the MM samples (Supplementary Table S2).

### A shift towards aerobic glycolytic metabolism in sPCL

**Figure 4:**
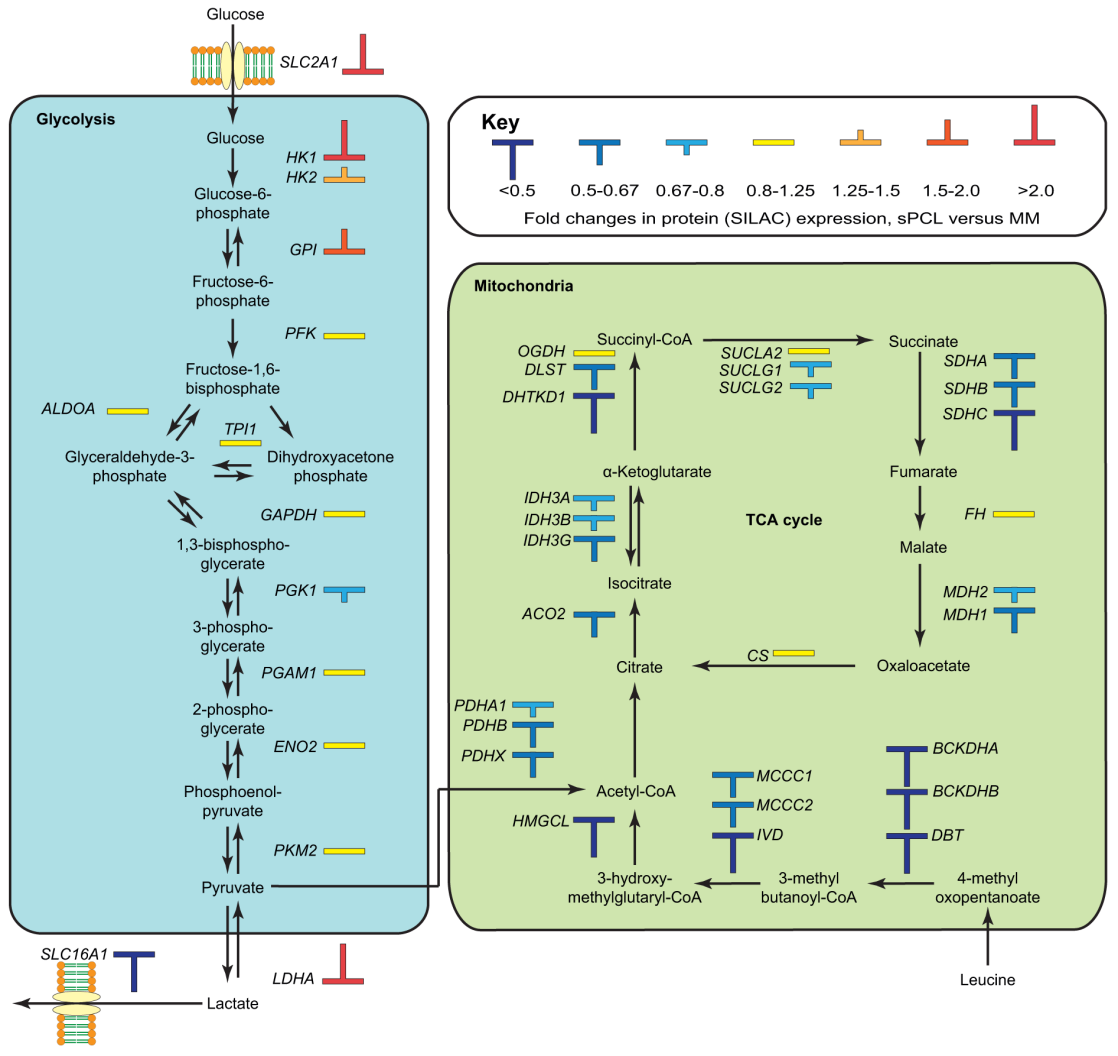
Overview of differentially expressed proteins in the glycolytic and oxidative metabolic pathways The observed up-regulation of factors in glycolytic glucose metabolism and down-regulation of factors in the mitochondrial oxidative metabolism conforms to an increased Warburg type metabolism in the sPCL cells.

The second most affected pathway in IPA was TCA cycle (p~1.7E-06), in which 8 out of 23 proteins (SDHA, SDHB, IDH3G, DHTKD1, ACO1, ACO2, DLST and MDH1) were differentially expressed, all of which were significantly down-regulated (Figure 4, Supplementary Table S2). This is in agreement with the observed overall down-regulation of mitochondrial proteins, and supports that mitochondrial oxidative metabolism is impaired in the sPCL cells. Two other mitochondrial pathways, leucine degradation and valine degradation were also among the top five most affected pathways according to IPA (Figure 3C). The degradation of these branched-chain amino acids as well as isoleucine takes place in the mitochondria and donates substrates to the TCA cycle. As illustrated in Figure 4 (Supplementary Table S2), nearly all enzymes involved in mitochondrial degradation of leucine were significantly down-regulated, and this was also observed for enzymes involved in valine degradation (Supplementary Table S2). Somewhat surprisingly, the level of the mitochondrial fatty acid transporter CPT1, the rate-limiting enzyme in mitochondrial fatty acid degradation, was 1.6-fold up-regulated in the sPCL cells. CPT1 activity is, however, inhibited by malonyl-CoA. In lipid-rich tissues including the bone marrow, hormones such as leptin inhibits formation of malonyl-CoA. Lower levels of adipokines in the bloodstream may thus contribute to inhibit CTP1 catalytic activity and mitochondrial fatty acid degradation in sPCL.

Down-regulation of mitochondrial respiration is commonly observed in tumor cells, with a concomitant increase in glycolytic metabolism. The Warburg effect was originally thought to be an adaption of cancer cells to the low oxygen tension in rapidly growing tumors [43]. However, the glycolytic metabolism of cancer cells is also observed under ample oxygen supply (aerobic glycolysis) and is often observed in leukemia [44, 45] and lung tumors [46, 47]. Thus the metabolic shift appears to be advantageous to the cancer cells via other pathways, which promote cell growth and survival [48] resistance to apoptosis [49] and contribute to chemotherapeutic drug resistance [29]. Although glycolysis was not among the pathways reported to be significantly affected in IPA, several key enzymes relevant to this pathway were found to be up-regulated, including the glucose importer SLC2A1 (GLUT-1, 3.8-fold, p~0.008), the regulatory hexokinase HK1 (3.5-fold, p~0.0001) and LDHA (2.2-fold, p~0.0001) (Figure 4). The latter is crucial to regenerate NAD^+^ to drive glycolytic ATP production. Targeting of glycolytic enzymes has emerged as an attractive strategy to selectively kill tumor cells, and several drugs are now in various phases of clinical trials [50]. Blocking glucose transporters such as SLC2A1 (GLUT-1) to inhibit glucose uptake can be mediated by compounds like WZB117 and fasentin and has been demonstrated to be selectively toxic to cancer cells harboring glycolytic metabolism [51, 52]. Inhibition of hexokinase is another attractive strategy since this is the first commitment step of glycolysis. Moreover, HK1 and HK2 bind to the outer mitochondrial membrane and may counteract mitochondrial death pathways as well as excessive mitochondrial ROS [53]. Here, the glucose analog and HK inhibitor 2-deoxy-D-glucose (2-DG) has demonstrated promising anticancer effects in preclinical models and was recently shown to sensitize acute lymphoblastic leukemia B-cells to Dasatinib treatment [54] and has been implemented in phase-I clinical trials in patients with advanced solid tumors [55]. Finally, inhibition of LDHA by a small drug like molecule (FX11) was shown to induce oxidative stress and cell death in human lymphoma B-cells, and was able to induce lymphoma regression when combined with the NAD^+^ synthesis inhibitor FK866 [56].

It is not likely that the apparent metabolic switch in the malignant cells at the sPCL stage compared to the MM stage is mediated by the change of microenvironment *per se*. There is a significantly higher mean oxygen tension in the peripheral blood (12%) compared to bone marrow (6.6%) [57], which should contribute to proteasomal degradation of the hypoxia-inducible transcription factors HIF-1 and HIF-2 ([58] and references therein) and thus decreased expression of e.g. SLC2A1, LDHA and HK1. We find, however, that these enzymes are >2-fold up-regulated in the sPCL cells, suggesting that the metabolic shift is advantageous to the cells via other pathways and potentially contributing to sPCL transformation. In summary, the above findings suggest that inhibitors targeting glycolytic enzymes should be explored as potential novel adjuvants in the treatment of sPCL when a glycolytic cancer phenotype is evident.

### Other factors potentially contributing to sPCL progression

In addition to the affected pathways as revealed by IPA analysis, several other proteins were found to be differentially expressed that potentially could contribute to progression from MM to sPCL. Table 1 highlights the 10 most up- and down-regulated proteins identified in the super-SILAC dataset. Here serum amyloid A4 (SAA4) protein was found to be the most up-regulated protein (21-fold) at the sPCL stage. SAA1/2 proteins are acute-phase proteins predominantly produced in the liver, but is also found in diseased tissues, including cancer cells [59]. Their potential involvement in carcinogenesis and tumor progression has been subject to considerable interest due to their ability to rapidly induce cytokine production and ROS [60], cell migration and proliferation [61]. Less is known about the SAA4 protein, but some studies have reported abundant expression of SAA4 in tumorigenic tissues [62–64] and recent findings assign a likely role of the protein during cellular invasion [65]. The strong induction of apolipoprotein D (APOD, 9.75-fold) in the sPCL samples was somewhat surprising. Several studies have reported an inverse correlation between APOD expression and cell proliferation [66] as well as invasive cancer phenotype [67]. However, recent findings indicate that APOD is up-regulated under oxidative stress and plays a catalytic role in inhibiting lipid peroxidation chain reactions [68, 69]. Such a function of APOD in sPCL would conform to the observed shift in metabolic enzymes towards aerobic glycolysis that mediates decreased ROS, and could indicate a combined attempt to reduce oxidative stress. This could be especially important during detachment of myeloma cells from the extracellular matrix (ECM) of the bone marrow. Detachment of cells from the ECM is known to induce a programmed cell death process named anoikis [70]. Although the mechanisms whereby cancer cells evade anoikis remain poorly understood, detachment from the ECM is accompanied by striking increases in ROS and increased ROS tolerance was recently shown to promote anchorage-independent growth of breast cancer cells [71].

**Table 1:** List of ten most up- and down-regulated proteins in sPCL versus MM based on the super-SILAC data

Gene	Protein	Fold change	P-value
SAA4	Serum amyloid A-4 protein	21.20	8.13E-05
Cd3APOD	Apolipoprotein D	9.75	0.00060
GBP2	Interferon-induced guanylate-binding protein 2	5.92	0.00030
S100A4	Protein S100-A4	5.52	0.00006
MKI67	Antigen KI-67	4.41	0.00152
TAGLN2	Transgelin-2	4.41	4.55E-07
ANXA3	Annexin A3	4.35	0.00055
DEK	Protein DEK	3.96	0.00016
TBCEL	Tubulin-specific chaperone cofactor E-like protein	3.70	0.03610
FLNA	Filamin-A	3.62	3.25E-07
IER3IP1	Immediate early response 3-interacting protein 1	0.161	0.03874
SERPINB6	Serpin B6	0.148	0.00036
FCER2	Low affinity immunoglobulin epsilon Fc receptor	0.136	0.00028
PLD4	Phospholipase D4	0.113	0.00096
RBP1	Retinol-binding protein 1	0.097	0.01287
CKB	Creatine kinase B-type	0.079	3.13E-05
SYPL1	Synaptophysin-like protein 1	0.077	0.00187
H1F0	Histone H1.0	0.061	0.00510
CRP	C-reactive protein	0.060	0.00047
MARCKS	Myristoylated alanine-rich C-kinase substrate	0.045	3.93E-05

GBP2 (5.92-fold up-regulated in sPCL) is a member of the interferon-induced guanylate family of GTPases. It has been shown to induce proliferation in mouse fibroblasts [72] and to regulate hematopoietic lineage differentiation [73]. S100A4 (5.52-fold up-regulated in sPCL), also known as metastasin, is a calcium-binding protein found in a wide range of cells, and that is involved in many cellular processes including proliferation, differentiation and tumor cell invasion [74]. Interestingly, it has also been shown to be involved in metabolic regulation by down-regulating mitochondrial respiration and activating glycolytic flux in malignant melanoma cells [75]. Treatment of the cells with dichloroacetate (DCA) reversed the glycolytic phenotype and preferentially induced apoptosis in the S100A4-stimulated cells. Although DCA does not directly inhibit glycolytic flux, it inhibits pyruvate dehydrogenase kinase, and thus increases the catalytic activity of pyruvate dehydrogenases and increases acetyl-CoA introduced into the TCA cycle. This increases ROS and reduces cytosolic regeneration of NAD^+^. The results from S100A-stimulated melanoma cells corroborate previous findings from our own laboratory in that DCA was selectively cytotoxic to melphalan-resistant myeloma cells with acquired Warburg metabolism [29]. Finally, increased proliferative potential of the sPCL cells was underscored by the 4.41-fold up-regulation of the proliferation-marker MKI67 (Ki-67) that is a marker for poor survival in multiple myeloma [76].

The most down-regulated protein in sPCL was MARCKS (22.2-fold). MARCKS plays an important role in cell adhesion, spreading and invasion of various tumor cells [77, 78] and elevated levels of the protein was recently associated with multidrug resistance in MM cell lines [79]. These findings are not easily reconciled with the strong down-regulation of MARCKS in sPCL. However, MARCKS may have other functions beyond regulating the actin cytoskeleton and cell motility. In BAF3 murine pro-B-cells MARCKS apparently has a role in ROS signaling [80]. To what degree its down-regulation mediates protection against oxidative stress in sPCL remains, however, to be established. C-reactive protein (CRP) was 16.7-fold down-regulated in the sPCL cells. This acute-phase protein is produced in the liver and strongly binds to the surface of dead and dying cells. We thus speculate that CRP detected in our cell samples originate from its binding to the surface of dying malignant plasma cells, which likely constitute a larger fraction of the malignant plasma cells in the bone marrow than in the highly proliferating sPCL cells. The 16.1-fold reduction in histone H1.0 (Table 1) and a concomitant 4.5-5.7-fold increase in the other histone H1 isoforms (Supplementary Table S2) strongly conform to a more dedifferentiated state in the sPCL cells [81] in agreement with the observed increase in proliferation and motility markers. Moreover, low levels of histone H1.0 has been suggested as a prognostic marker for poor survival of patients with malignant gliomas [82]. Creatine kinase B (CKB, 12.7-fold down-regulated) has been found to be secreted by metastatic cells, potentially to convert extracellular creatine to phosphocreatine that can be imported and serve as an ATP-generating source and promote metastatic survival [83]. It is thus likely that the down-regulated expression of CKB is a result of the migration of the malignant plasma cells to a less hypoxic environment, and not a mediator of bone marrow evasion *per se*. The retinol-binding protein RBP1 (10.3-fold down-regulated in sPCL) is a tumor suppressor protein that is involved in the intracellular retinoic acid (RA) metabolism and treatment with RA inhibitors has been shown to inhibit MM cell growth and to induce apoptosis [84]. Moreover, RBP1 is epigenetically inactivated in many cases of MM and is associated with an unfavorable prognosis [85]. The low-affinity immunoglobulin epsilon Fc factor FCER2 (CD23) was 7.4-fold down-regulated in sPCL. Surface expression of CD23 has previously been observed in a subclass of MM with abnormalities at chromosome 11 and has been associated with primary PCL in this subgroup [86]. The clinical relevance of CD23 in this subgroup however remains to be established. CD23 exists in both a membrane-bound and soluble form. The soluble form originates from shedding of membrane bound CD23 and displays pleotropic biological activities, including inhibition of apoptosis of germinal center B-cells [87] and proliferation of myeloid precursor cells [88]. Shedding of CD23 from MM cells has been shown to be induced by a disintegrin and metalloprotease ADAM10 [89]. Notably, we found that ADAM10 was 2.2-fold (p ~ 0.007) in sPCL (Supplementary Table S2), in accordance with the reduced levels of cellular CD23.

### Differential protein expression was not accompanied by altered overall genomic methylation or hydroxymethylation

The relatively large number of differentially expressed proteins (795) in the malignant plasma cells from the MM and sPCL stages in the same patient was somewhat surprising and suggested the involvement of global epigenetic alterations. Moreover, among the differentially expressed proteins, the majority (64%, 505 proteins) were down-regulated. Aberrant methylation of cytosine residues in the promotor regions is associated with transcriptional silencing [90] and epigenetic inactivation of tumor suppressor genes has been linked to altered microenvironment pathways and an unfavorable prognosis in multiple myeloma [85]. Moreover, we observed a 2.3-fold increase in the maintenance DNA methyltransferase DNMT1 at the sPCL stage in our dataset (Supplementary Table S2). Progressively increasing DNMT1 mRNA expression has previously been reported in a study encompassing healthy donors, MM and PCL [91]. To analyze whether aberrant cytosine methylation was involved in the transformation to sPCL, we subjected DNA samples from the MM and sPCL stages to mass-spectrometry based overall 5-methylcytosine (5-mC) quantification. We also included quantification of 5-hydroxymethylcytosine (5-OHmC) in the analyses, since it is an important intermediate in epigenetic reprogramming of 5-mC [92–94]. The analyses revealed no significant change in 5-mC at the MM and sPCL stages (0.72% and 0.76% of total deoxynucleosides, respectively). A moderate, but non-significant decrease was observed in global 5-OHmC (3.2 versus 2.7 residues per 10^6^ deoxynucleosides in MM and sPCL, respectively). These results indicate that aberrant methylation is not a major cause of the significant shift in protein expression during progression to sPCL. Thus the increased expression in DNMT1 rather reflects increased proliferation at the sPCL stage, in agreement with previous studies in human cancers showing that DNMT1 is proliferation dependent [95, 96].

### Concluding remarks

In summary, we present the first comprehensive protein profile highlighting differentially expressed proteins accompanying progression of MM to sPCL in a single patient. Many of these are amenable to targeting by small molecule inhibitors currently approved for clinical use. Further studies must be undertaken to verify whether our findings represent a common phenotype in the progression of MM to sPCL, or to what extent this holds true for subgroups harboring specific genetic alterations. In this respect, our super-SILAC library can be a valuable tool for other research groups conducting quantitative proteome profiling in MM and sPCL.

## MATERIALS AND METHODS

### Preparation of a super-SILAC multiple myeloma library

Six human multiple myeloma- and two human B-cell lymphoma cell lines were used to generate a heavy super-SILAC library for quantitative proteome profiling. The multiple myeloma cell lines IH-1 [97], OH-2 [25], KJON and VOLIN [26] were established in the laboratory of the Myeloma group at the Department of Cancer Research and Molecular Medicine, NTNU. The human multiple myeloma cell line INA-6 [98] was a kind gift from Dr M. Gramatzki (University of Erlangen-Nurnberg, Erlangen, Germany). RPMI8226-LR5 [99] was kindly supplied by Prof. William S. Dalton at the H. Lee Moffitt Cancer Center & Research Institute, Tampa, USA. The germinal center B cell-like DLBCL cell line KARPAS-422 [100] was obtained from DSMZ (Braunschweig, Germany) and the human Burkitt`s lymphoma cell line RAMOS [101] was obtained from ATCC. None of these cell lines was reported to be mis-identified or contaminated according to the International Cell Line Authentication Committee (http://iclac.org/databases/cross-contaminations).

All cell lines were passaged twice weekly in SILAC medium consisting of RPMI-1640 (Sigma-Aldrich, Germany) medium with ^13^C_6_^15^N_2_-lysine (K8; ^13^C_6_ 99%, ^15^N_2_ 99%) and ^13^C_6_^15^N_4_-arginine (R10;^13^C_6_99%, ^15^N_4_ 99%) (Cambridge Isotope Laboratories, Andover, MA) instead of the natural amino acids, dialyzed fetal bovine or human serum as appropriate, L-glutamine (100 μg/mL) and gentamicin (20 μg/mL). For IL-6 dependent cell lines (OH-2, VOLIN, KJON, IH-1, INA-6) 1 ng/mL recombinant human interleukin IL-6 (Biosource, Camarillo, CA, USA) was added. The Melphalan-resistant RPMI8226-LR5 was maintained under constant selection through the addition of 1 μM melphalan (Sigma-Aldrich, St. Louis, MO) twice weekly. Cells were cultured for at least eight passages in the SILAC medium at 37°C in a humidified atmosphere containing 5% CO_2_ in order to accomplish near complete labeling. Incorporation was examined by separate quantitative LC-MS/MS analysis and equal amounts of protein from each of the heavy lysates were premixed to generate the super-SILAC mix.

### Patient material

At diagnosis her disease was characterized by Hgb 11.3 mg/dl, M protein of IgG-kappa type 20,5 g/l, 62% plasma cells in the bone marrow, multiple osteolytic lesions, ISS stage 2. t(11;14) was detected whereas t(4;14), del 13 and del 17 were negative by FISH. During the initial four years she was sensitive to induction treatment with cyclophosphamide/bortezomib/dexamethasone followed by high dose melphalan with autologous stem cell transplantation (very good partial remission), bortezomib/melphalan/prednisone (partial remission) and cyclophosphamide/prednisone (partial remission) and resistant to thalidomide/prednisone and lenalidomide/prednisone. After four years, plasma cell leukemia was diagnosed with 35% plasma cells in blood smears. She was then resistant to all drugs and died after two months. Malignant CD138^+^ cells were stored in DMSO at -80°C in the Norwegian Myeloma Biobank at St. Olav's Hospital prior to analysis. The study was approved by the Regional committee for medical and health research ethics in Mid-Norway (REK 2011/2029, REK 2012/1501) and conforms to the Declaration of Helsinki

### Sample preparation for mass spectrometry

A bone marrow sample was collected prior to the first treatment and blood samples were collected when sPCL had developed four years later. Plasma cells were purified by CD138+ magnetic-activated cell sorting (MACS) microbeads (Miltenyi, CA). For MM samples, two aliquots of CD138^+^ cells were individually isolated and each subjected to four technical MS replicate analyses. At the time of sPCL diagnosis, six blood samples were collected over a time span of four days, each from which two aliquots of CD138^+^ cells were individually isolated and each subjected to two technical MS replicate analyses.

We employed methanol/chloroform [102] protein precipitation method with modifications. In short, 500 000 cells were homogenized in 25 μL 7 M urea, 2 M thiourea, 2.5% CHAPS, 25 mM DTT using Kontes™ Pellet Pestle™ Motor. After mixing and thorough vortexing cells were incubated for 15 minutes at room temperature to solubilize proteins and the homogenate was clarified by centrifugation at 16 000 × g for 15 min at room temperature. 25 μg protein from each patient sample was mixed 1:1 with super-SILAC protein standard. For protein precipitation, the following (all volumes refer to the volume of the original patient sample/super-SILAC mixture) were stepwise added with vortexing following each addition: Four volumes of methanol, one volume chloroform and three volumes of distilled water. The sample was centrifuged at 14 000 × g for 2 min at room temperature and the upper aqueous phase carefully removed. Four volumes of methanol were then added and vortexed and the mixture centrifuged at 14 000 × g for 2 min at room temperature. The supernatant was carefully removed and 100 μl trypsin (10 ng/μL in aqueous 50 mM ammonium bicarbonate) was added directly to the protein pellet. The sample was incubated at 37°C with shaking at 600 rpm for 16 h, evaporated to dryness and dissolved in 0,1% formic acid. The resulting solution was centrifuged at 16 000 × g for 1 min and the supernatant subjected to MS analysis.

### Mass spectrometry and analysis of super-SILAC data

Peptides were analyzed on a LC-MS/MS platform consisting of an Easy-nLC 1000 UHPLC system (Thermo Scientific/Proxeon) interfaced with an LTQ-Orbitrap Elite hybrid mass spectrometer (Thermo Scientific) via a nanospray ESI ion source (Proxeon, Odense). Peptides were injected onto a C-18 trap column (Acclaim PepMap100, 75 μm i. d. x 2 cm, C18, 5 μm, 100 Å, Thermo Scientific) and further separated on a C-18 analytical column (Acclaim PepMap100, 75 μm i. d. x 50 cm, C18, 3 μm, 100 Å, Thermo Scientific). The LC was operated at 250 nL/min over 262 min with solvent A consisting of 0.1% formic acid in water and solvent B of 0.1% formic acid in CH_3_CN. Peptides were eluted with a linear gradient of 0-30% solvent B over 252 min, followed by 5 min at 100% B and 5 min at 100% A. Peptides were analyzed on the mass spectrometer operating in positive ion- and data dependent acquisition (DDA) mode using the following parameters: Electrospray voltage 2.2 kV, CID fragmentation with normalized collision energy 35, automatic gain control (AGC) target value of 1E6 for Orbitrap MS and 1E4 for MS/MS scans. Each MS scan (m/z 400–1600) was acquired at a resolution of 120,000 FWHM, followed by 20 MS/MS scans triggered for intensities above 500 and selected with an isolation window of 2 Th, at a maximum ion injection time of 200 ms for MS and 50 ms for MS/MS scans. Raw files were analyzed with MaxQuant v 1.5 [103] using its default settings with multiplicity 2 (Arg10,Lys8), FTMS and ITMS MS/MS tolerance of 0.5 Da and 20 ppm, respectively. Search was performed against the June 2014 version of Human proteome set with isoforms from Uniprot [104]. Values from technical replicates were transformed to log_2_ in order to have a better approximation to normal distribution of super-SILAC ratios. Further, the median of technical replicates were calculated to represent the samples in order to be less sensitive to outliers. These are presented in in Supplementary Table S2 from column V to AA representing three time points for MM and sPCL stages respectively.

The mass spectrometry proteomics data have been deposited to the ProteomeXchange Consortium [105] via the PRIDE partner repository with the dataset identifier PXD001963.

## SUPPLEMENTARY MATERIALS FIGURES AND TABLES






