# Supplementary material for: Proteome alterations associated with transformation of multiple myeloma to secondary plasma cell leukemia
Source: Oncotarget. 2016 Dec 27;8(12):19427–42. doi: 10.18632/oncotarget.14294 (PMC5386695; doi:10.18632/oncotarget.14294)
Supplement: Supplementary file 1 [file oncotarget-08-19427-s001.pdf]

Proteome alterations associated with transformation of multiple myeloma to secondary plasma cell leukemia

SUPPLEMENTARY FIGURES

Supplementary Table 1: Complete list of pairwise correlation coefficients and P-values for the global expression patterns in the super-SILAC library.

See Supplementary File 1

Supplementary Table 2: Summarized MaxQuant report for sPCL versus multiple myeloma.

See Supplemenatary File 2
